# Supplementary material for: Efficacy of Bifidobacterium animalis subsp. lactis BL-99 in the treatment of functional dyspepsia: a randomized placebo-controlled clinical trial
Source: Nat Commun. 2024 Jan 3;15:227. doi: 10.1038/s41467-023-44292-x (PMC10764899; doi:10.1038/s41467-023-44292-x)
Supplement: Supplementary file 1 — Supplementary information [file 41467_2023_44292_MOESM1_ESM.pdf]

Contents

1. Supplementary Tables 1

Supplementary Table 1 The clinical response rate for all (men and women) participants based on per-protocol (PP) set 1

Supplementary Table 2 The clinical response rate for men participants based on intention-to-treat (ITT) set 2

Supplementary Table 3 The clinical response rate for women participants based on intention-to-treat (ITT) set 3

Supplementary Table 4 The clinical response rate in participants with BMI < 24 kg/m^2 based on intention-to-treat (ITT) set 4

Supplementary Table 5 The clinical response rate in participants with BMI ≥ 24 kg/m^2 based on intention-to-treat (ITT) set 5

Supplementary Table 6 No symptoms after treatment for all (men and women) participants based on intention-to-treat (ITT) set 6

Supplementary Table 7 Symptom score for men and women participants based on Intention-to-Treat (ITT) set 7

Supplementary Table 8 Symptom score for men and women participants based on Per-Protocol (PP) Set 8

Supplementary Table 9 Adverse events 9

Supplementary Table 10 Gene information related to the adhesion function of probiotics 10

2. Supplementary Figures 11

Supplementary Fig. 1. Distance-based redundancy analysis (dbRDA) of microbiota communities in the fecal samples among four groups at baseline and post treatment period. 11

Supplementary Fig. 2. Composition of the gut microbiota of samples at baseline and post treat period. 12

Supplementary Fig. 3. Changes of species from the baseline to the post treatment period. For each species, the left four panels show the changes in relative abundances between the baseline and post treatment period in four groups. 13

Supplementary Fig. 4. Principal coordinates analysis (PCoA) of fecal metabolome among four groups at baseline and post treatment period. 14

Supplementary Fig. 5. Effect of short-chain fatty acid infusion on serum gastrin. 15

Supplementary Fig. 6 The 16s gene map of Bifidobacterium animalis subsp. lactis. BL-99. 16

Supplementary Fig. 7. The study design of the trial 17
3. Supplementary Methods 18
3.1 Metagenomic Analysis 18
3.2 Non-target metabolic 18
3.3 Short Chain Fatty Acids (SCFAs) Measures 19
3.4 SCFA infusion experiment 19
3.4.1 Ethics statement 19
3.4.2 Animals 19
3.4.3 Intracarotid SCFA infusion experiments 19
3.4.4 Statistical analysis 20
4. Supplementary Note1: FD symptom questionnaire 21
5. Supplementary Note2: Study protocol 24
6. Supplementary Note3: Statistical analysis plan 29
Supplementary Reference 38

1. Supplementary Tables

Supplementary Table 1 The clinical response rate for all (men and women) participants based on per-protocol (PP) set

Clinical response rate[*)], No. (%)		Placebo (n=45)	Positive_control (n=48)	BL-99_low (n=47)	BL-99_high (n=45)	$p_{overall}$	*p*					
							Positive_control *vs* Placebo	BL-99_low *vs* Placebo	BL-99_high *vs* Placebo	BL-99_low *vs* Positive_control	BL-99_high *vs* Positive_control	BL-99_high *vs* BL-99_low
4-week treatment	FD score [a)]	28 (62.2)	27 (56.3)	27 (57.4)	36 (80.0)	0.067	-	-	-	-	-	-
	PDS score [b)]	30 (66.7)	31 (64.6)	35 (74.5)	41 (91.1)	0.016	0.833	0.412	0.008	0.297	0.004	0.043
	EPS score [c)]	24 (53.3)	22 (45.8)	24 (51.1)	33 (73.3)	0.044	0.470	0.828	0.051	0.610	0.008	0.030
8-week treatment	FD score	28 (62.2)	34 (70.8)	36 (76.6)	43 (95.6)	0.002	0.38	0.137	0.001	0.524	0.006	0.019
	PDS score	33 (73.3)	38 (79.2)	38 (80.9)	42 (93.3)	0.094	-	-	-	-	-	-
	EPS score	24 (53.3)	26 (54.2)	30 (63.8)	35 (77.8)	0.049	0.936	0.308	0.016	0.339	0.019	0.145
2-week follow-up	FD score	30 (66.7)	32 (66.7)	37 (78.7)	40 (88.9)	0.038	1.000	0.197	0.015	0.190	0.014	0.194
	PDS score	35 (77.8)	37 (77.1)	42 (89.4)	42 (93.3)	0.071	0.936	0.140	0.047	0.117	0.039	0.503
	EPS score	26 (57.8)	29 (60.4)	31 (66.0)	36 (80.0)	0.114	-	-	-	-	-	-
8-week questionnaire survey	FD score	9 (20.0)	7 (14.6)	8 (17.0)	14 (31.1)	0.213	-	-	-	-	-	-
	PDS score	11 (24.4)	15 (31.3)	16 (34.0)	17 (37.8)	0.577	-	-	-	-	-	-
	EPS score	5 (11.1)	8 (16.7)	5 (10.6)	13 (28.9)	0.047	0.443	0.942	0.041	0.400	0.163	0.033

[*)] Clinical response rate was defined as the proportion of participants with a score (i.e., FD score, PDS score, and EPS score) decrease >0.5.

[a)]FD score: the composite functional dyspepsia score is calculated as the mean of postprandial fullness, early satiety, epigastric pain, and epigastric burning scores. [b)]PDS score: the postprandial distress syndrome score is calculated as the mean of postprandial fullness score and early satiety score. [c)]EPS score: the epigastric pain syndrome score is calculated as the mean of epigastric pain score and epigastric burning score.

All hypothesis tests were two-sided. $p < 0.05$ was considered significant.

BL-99, *Bifidobacterium animalis* subsp. *lactis* BL-99.

Patients in the placebo, positive_control, BL-99_low, and BL-99_high groups were administered with maltodextrin (2 g/day), rabeprazole (10 mg/ day), low-dose BL-99 (1×10^{10} CFU/day), and high-dose BL-99 (5×10^{10} CFU/day), respectively. Source data are provided as a Source Data file.

Supplementary Table 2 The clinical response rate for men participants based on intention-to-treat (ITT) set

Clinical response rate[*], No. (%)		Placebo (n=13)	Positive_control (n=12)	BL-99_low (n=13)	BL-99_high (n=13)	*p* overall	*p*					
							Positive_control *vs* Placebo	BL-99_low *vs* Placebo	BL-99_high *vs* Placebo	BL-99_low *vs* Positive_control	BL-99_high *vs* Positive_control	BL-99_high *vs* BL-99_low
4-week	FD score [a]	6 (46.2)	9 (75.0)	9 (69.2)	11 (84.6)	0.185	-	-	-	-	-	-
treatment	PDS score [b]	7 (53.8)	8 (66.7)	11 (84.6)	13 (100.0)	0.032	0.515	0.102	0.998	0.303	0.999	0.999
	EPS score [c]	9 (69.2)	8 (66.7)	9 (69.2)	11 (84.6)	0.729	-	-	-	-	-	-
8-week	FD score	10 (76.9)	9 (75.0)	9 (69.2)	13 (100.0)	0.211	-	-	-	-	-	-
treatment	PDS score	11 (84.6)	10 (83.3)	10 (76.9)	13 (100.0)	0.370	-	-	-	-	-	-
	EPS score	9 (69.2)	8 (66.7)	8 (61.5)	11 (84.6)	0.605	-	-	-	-	-	-
2-week	FD score	8 (61.5)	9 (75.0)	9 (69.2)	13 (100.0)	0.111	-	-	-	-	-	-
follow-up	PDS score	9 (69.2)	9 (75.0)	10 (76.9)	13 (100.0)	0.211	-	-	-	-	-	-
	EPS score	9 (69.2)	10 (83.3)	9 (69.2)	13 (100.0)	0.152	-	-	-	-	-	-
8-week	FD score	5 (38.5)	6 (50.0)	1 (7.7)	5 (38.5)	0.130	-	-	-	-	-	-
questionnaire	PDS score	5 (38.5)	5 (41.7)	3 (23.1)	6 (46.2)	0.644	-	-	-	-	-	-
survey	EPS score	3 (23.1)	4 (33.3)	1 (7.7)	6 (46.2)	0.160	-	-	-	-	-	-

Note: [*] Clinical response rate was defined as the proportion of participants with a score (i.e., FD score, PDS score, and EPS score) decrease >0.5.

[a]FD score: the composite functional dyspepsia score is calculated as the mean of postprandial fullness, early satiety, epigastric pain, and epigastric burning scores. [b]PDS score: the postprandial distress syndrome score is calculated as the mean of postprandial fullness score and early satiety score. [c]EPS score: the epigastric pain syndrome score is calculated as the mean of epigastric pain score and epigastric burning score.

All hypothesis tests were two-sided. $p < 0.05$ was considered significant.

BL-99, *Bifidobacterium animalis* subsp. *lactis* BL-99.

Patients in the placebo, positive_control, BL-99_low, and BL-99_high groups were administered with maltodextrin (2 g/day), rabeprazole (10 mg/ day), low-dose BL-99 (1×10^{10} CFU/day), and high-dose BL-99 (5×10^{10} CFU/day), respectively. Source data are provided as a Source Data file.

Supplementary Table 3 The clinical response rate for women participants based on intention-to-treat (ITT) set

Clinical response rate[*], No. (%)		Placebo (n=37)	Positive_control (n=38)	BL-99_low (n=37)	BL-99_high (n=37)	p overall	p					
							Positive_control *vs* Placebo	BL-99_low *vs* Placebo	BL-99_high *vs* Placebo	BL-99_low *vs* Positive_control	BL-99_high *vs* Positive_control	BL-99_high *vs* BL-99_low
4-week treatment	FD score [a)]	23 (62.2)	19 (50.0)	19 (51.4)	27 (73.0)	0.151	-	-	-	-	-	-
	PDS score [b)]	24 (64.9)	24 (63.2)	26 (70.3)	30 (81.1)	0.326	-	-	-	-	-	-
	EPS score [c)]	15 (40.5)	15 (39.5)	16 (43.2)	24 (64.9)	0.092	-	-	-	-	-	-
8-week treatment	FD score	19 (51.4)	26 (68.4)	28 (75.7)	32 (86.5)	0.009	0.134	0.032	0.002	0.485	0.068	0.241
	PDS score	23 (62.2)	29 (76.3)	30 (81.1)	31 (83.8)	0.132	-	-	-	-	-	-
	EPS score	15 (40.5)	19 (50.0)	23 (62.2)	26 (70.3)	0.051	-	-	-	-	-	-
2-week follow-up	FD score	23 (62.2)	24 (63.2)	29 (78.4)	29 (78.4)	0.219	-	-	-	-	-	-
	PDS score	27 (73.0)	29 (76.3)	34 (91.9)	31 (83.8)	0.160	-	-	-	-	-	-
	EPS score	17 (45.9)	20 (52.6)	23 (62.2)	25 (67.6)	0.238	-	-	-	-	-	-
8-week questionnaire survey	FD score	5 (13.5)	2 (5.3)	8 (21.6)	11 (29.7)	0.035	0.235	0.363	0.097	0.053	0.012	0.426
	PDS score	7 (18.9)	11 (28.9)	15 (40.5)	13 (35.1)	0.214	-	-	-	-	-	-
	EPS score	2 (5.4)	5 (13.2)	5 (13.5)	9 (24.3)	0.136	-	-	-	-	-	-

Note: [*)] Clinical response rate was defined as the proportion of participants with a score (i.e., FD score, PDS score, and EPS score) decrease >0.5.

[a)]FD score: the composite functional dyspepsia score is calculated as the mean of postprandial fullness, early satiety, epigastric pain, and epigastric burning scores. [b)]PDS score: the postprandial distress syndrome score is calculated as the mean of postprandial fullness score and early satiety score. [c)]EPS score: the epigastric pain syndrome score is calculated as the mean of epigastric pain score and epigastric burning score.

All hypothesis tests were two-sided. $p < 0.05$ was considered significant.

BL-99, *Bifidobacterium animalis* subsp. *lactis* BL-99.

Patients in the placebo, positive_control, BL-99_low, and BL-99_high groups were administered with maltodextrin (2 g/day), rabeprazole (10 mg/ day), low-dose BL-99 (1×10^{10} CFU/day), and high-dose BL-99 (5×10^{10} CFU/day), respectively. Source data are provided as a Source Data file.

Supplementary Table 4 The clinical response rate in participants with BMI < 24 kg/m^2 based on intention-to-treat (ITT) set

Clinical response rate[*)], No. (%)		Placebo (n=19)	Positive_control (n=22)	BL-99_low (n=20)	BL-99_high (n=19)	p overall	p					
							Positive_control *vs* Placebo	BL-99_low *vs* Placebo	BL-99_high *vs* Placebo	BL-99_low *vs* Positive_control	BL-99_high *vs* Positive_control	BL-99_high *vs* BL-99_low
4-week treatment	FD score [a)]	11 (57.9)	11 (50.0)	11 (55.0)	14 (73.7)	0.464	-	-	-	-	-	-
	PDS score [b)]	12 (63.2)	13 (59.1)	16 (80.0)	17 (89.5)	0.106	-	-	-	-	-	-
	EPS score [c)]	8 (42.1)	8 (36.4)	8 (40.0)	14 (73.7)	0.072	-	-	-	-	-	-
8-week treatment	FD score	8 (42.1)	16 (72.7)	15 (75.0)	16 (84.2)	0.030	0.051	0.041	0.011	0.867	0.381	0.480
	PDS score	9 (47.4)	17 (77.3)	16 (80.0)	16 (84.2)	0.043	0.053	0.039	0.022	0.830	0.578	0.732
	EPS score	7 (36.8)	11 (50.0)	13 (65.0)	14 (73.7)	0.101	-	-	-	-	-	-
2-week follow-up	FD score	11 (57.9)	12 (54.5)	16 (80.0)	16 (84.2)	0.094	-	-	-	-	-	-
	PDS score	15 (78.9)	16 (72.7)	19 (95.0)	18 (94.7)	0.106	-	-	-	-	-	-
	EPS score	7 (36.8)	10 (45.5)	13 (65.0)	14 (73.7)	0.078	-	-	-	-	-	-
8-week questionnaire survey	FD score	2 (10.5)	2 (9.1)	4 (20.0)	3 (15.8)	0.730	-	-	-	-	-	-
	PDS score	2 (10.5)	9 (40.9)	7 (35.0)	5 (26.3)	0.164	-	-	-	-	-	-
	EPS score	0 (0.0)	2 (9.1)	2 (10.0)	2 (10.5)	0.562	-	-	-	-	-	-

Note: [*)] Clinical response rate was defined as the proportion of participants with a score (i.e., FD score, PDS score, and EPS score) decrease >0.5.

[a)]FD score: the composite functional dyspepsia score is calculated as the mean of postprandial fullness, early satiety, epigastric pain, and epigastric burning scores. [b)]PDS score: the postprandial distress syndrome score is calculated as the mean of postprandial fullness score and early satiety score. [c)]EPS score: the epigastric pain syndrome score is calculated as the mean of epigastric pain score and epigastric burning score.

All hypothesis tests were two-sided. $p < 0.05$ was considered significant.

BL-99, *Bifidobacterium animalis* subsp. *lactis* BL-99.

Patients in the placebo, positive_control, BL-99_low, and BL-99_high groups were administered with maltodextrin (2 g/day), rabeprazole (10 mg/ day), low-dose BL-99 (1×10^{10} CFU/day), and high-dose BL-99 (5×10^{10} CFU/day), respectively. Source data are provided as a Source Data file.

Supplementary Table 5 The clinical response rate in participants with BMI ≥ 24 kg/m² based on intention-to-treat (ITT) set

Clinical response rate[*], No. (%)		Placebo (n=31)	Positive_control (n=28)	BL-99_low (n=30)	BL-99_high (n=31)	p overall	p					
							Positive_control *vs* Placebo	BL-99_low *vs* Placebo	BL-99_high *vs* Placebo	BL-99_low *vs* Positive_control	BL-99_high *vs* Positive_control	BL-99_high *vs* BL-99_low
4-week treatment	FD score [a)]	18 (58.1)	17 (60.7)	17 (56.7)	24 (77.4)	0.299	-	-	-	-	-	-
	PDS score [b)]	19 (61.3)	19 (67.9)	21 (70.0)	26 (83.9)	0.256	-	-	-	-	-	-
	EPS score [c)]	16 (51.6)	15 (53.6)	17 (56.7)	21 (67.7)	0.582	-	-	-	-	-	-
8-week treatment	FD score	21 (67.7)	19 (67.9)	22 (73.3)	29 (93.5)	0.058	0.992	0.633	0.019	0.647	0.021	0.048
	PDS score	25 (80.6)	22 (78.6)	24 (80.0)	28 (90.3)	0.611	-	-	-	-	-	-
	EPS score	17 (54.8)	16 (57.1)	18 (60.0)	23 (74.2)	0.400	-	-	-	-	-	-
2-week follow-up	FD score	20 (64.5)	21 (75.0)	22 (73.3)	26 (83.9)	0.384	-	-	-	-	-	-
	PDS score	21 (67.7)	22 (78.6)	25 (83.3)	26 (83.9)	0.384	-	-	-	-	-	-
	EPS score	19 (61.3)	20 (71.4)	19 (63.3)	24 (77.4)	0.500	-	-	-	-	-	-
8-week questionnaire survey	FD score	8 (25.8)	6 (21.4)	5 (16.7)	13 (41.9)	0.131	-	-	-	-	-	-
	PDS score	10 (32.3)	7 (25.0)	11 (36.7)	14 (45.2)	0.427	-	-	-	-	-	-
	EPS score	5 (16.1)	7 (25.0)	4 (13.3)	13 (41.9)	0.039	0.401	0.759	0.030	0.264	0.174	0.017

Note: [*)] Clinical response rate was defined as the proportion of participants with a score (i.e., FD score, PDS score, and EPS score) decrease >0.5.

[a)]FD score: the composite functional dyspepsia score is calculated as the mean of postprandial fullness, early satiety, epigastric pain, and epigastric burning scores. [b)]PDS score: the postprandial distress syndrome score is calculated as the mean of postprandial fullness score and early satiety score. [c)]EPS score: the epigastric pain syndrome score is calculated as the mean of epigastric pain score and epigastric burning score.

All hypothesis tests were two-sided. $p < 0.05$ was considered significant.

BL-99, *Bifidobacterium animalis* subsp. *lactis* BL-99.

Patients in the placebo, positive_control, BL-99_low, and BL-99_high groups were administered with maltodextrin (2 g/day), rabeprazole (10 mg/ day), low-dose BL-99 (1×10^{10} CFU/day), and high-dose BL-99 (5×10^{10} CFU/day), respectively. Source data are provided as a Source Data file.

Supplementary Table 6 No symptoms after treatment for all (men and women) participants based on intention-to-treat (ITT) set

No symptoms No. (%)		Placebo (n=50)	Positive_control (n=50)	BL-99_low (n=50)	BL-99_high (n=50)	$p_{overall}$	*p*					
							Positive_control *vs* Placebo	BL-99_low *vs* Placebo	BL-99_high *vs* Placebo	BL-99_low *vs* Positive_control	BL-99_high *vs* Positive_control	BL-99_high *vs* BL-99_low
4-week treatment	FD score [a)]	18 (36.0)	13 (26.0)	16 (32.0)	21 (42.0)	0.431	-	-	-	-	-	-
	PDS score [b)]	21 (42.0)	21 (42.0)	22 (44.0)	26 (52.0)	0.280	-	-	-	-	-	-
	EPS score [c)]	34 (68.0)	25 (50.0)	27 (54.0)	30 (60.0)	0.106	-	-	-	-	-	-
8-week treatment	FD score	21 (42.0)	18 (36.0)	29 (58.0)	28 (56.0)	0.554	-	-	-	-	-	-
	PDS score	24 (48.0)	25 (50.0)	30 (60.0)	34 (68.0)	0.680	0.841	0.230	0.044	0.029	0.046	0.840
	EPS score	36 (72.0)	30 (60.0)	38 (76.0)	34 (68.0)	0.471	-	-	-	-	-	-
2-week follow-up	FD score	18 (36.0)	21 (42.0)	36 (72.0)	39 (78.0)	0.004	0.539	<0.001	<0.001	0.03	<0.001	0.489
	PDS score	21 (42.0)	22 (44.0)	40 (80.0)	39 (78.0)	<0.001	0.840	<0.001	<0.001	<0.001	0.001	0.806
	EPS score	30 (60.0)	35 (70.0)	37 (74.0)	40 (80.0)	0.129	0.296	0.139	0.032	0.656	0.251	0.477
8-week questionnaire survey	FD score	0 (0.0)	1 (2.0)	0 (0.0)	0 (0.0)	0.201	-	-	-	-	-	-
	PDS score	0 (0.0)	1 (2.0)	0 (0.0)	0 (0.0)	0.347	-	-	-	-	-	-
	EPS score	2 (4.0)	1 (2.0)	0 (0.0)	0 (0.0)	0.186	-	-	-	-	-	-

Note: [*)] No symptoms: Patients who had symptom resolution (no symptoms) after 4-week treatment, 8-week treatment, 2-week follow-up or 8-week questionnaire survey.

[a)]FD score: the composite functional dyspepsia score is calculated as the mean of postprandial fullness, early satiety, epigastric pain, and epigastric burning scores. [b)]PDS score: the postprandial distress syndrome score is calculated as the mean of postprandial fullness score and early satiety score. [c)]EPS score: the epigastric pain syndrome score is calculated as the mean of epigastric pain score and epigastric burning score.

All hypothesis tests were two-sided. $p < 0.05$ was considered significant.

BL-99, *Bifidobacterium animalis* subsp. *lactis* BL-99.

Patients in the placebo, positive_control, BL-99_low, and BL-99_high groups were administered with maltodextrin (2 g/day), rabeprazole (10 mg/ day), low-dose BL-99 (1×10^{10} CFU/day), and high-dose BL-99 (5×10^{10} CFU/day), respectively. Source data are provided as a Source Data file.

Supplementary Table 7 Symptom score for men and women participants based on Intention-to-Treat (ITT) set

Symptom score		Men and women participants					Men participants					Women participants				
		Placebo (n=50)	Positive_control (n=50)	BL-99 low (n=50)	BL-99 high (n=50)	p overall	Placebo (n=13)	Positive_control (n=12)	BL-99 low (n=13)	BL-99 high (n=13)	p overall	Placebo (n=37)	Positive_control (n=38)	BL-99 low (n=37)	BL-99 high (n=37)	p overall
Baseline	FD score [a)]	1.60±0.87	1.61±0.61	1.62±0.75	1.86±0.67	0.087	1.90±0.95	1.94±0.68	1.50±0.83	2.00±0.73	0.161	1.50±0.82	1.51±0.56	1.67±0.73	1.83±0.65	0.056
	PDS score [b)]	1.92±0.79	1.95±0.47	1.95±0.75	2.10±0.69	0.234	2.12±0.77	2.04±0.62	1.69±0.97	2.15±0.72	0.150	1.85±0.78	1.92±0.41	2.04±0.73	2.08±0.68	0.179
	EPS score [c)]	1.28±1.10	1.27±0.95	1.29±0.95	1.65±0.83	0.072	1.69±1.25	1.83±0.81	1.31±0.97	1.85±0.83	0.215	1.14±1.03	1.09±0.93	1.28±0.95	1.58±0.84	0.053
4-week	FD score	0.59±0.68	0.76±0.66	0.64±0.58	0.58±0.69	0.055	0.56±0.61	0.79±0.78	0.31±0.43	0.40±0.72	0.146	0.59±0.71	0.77±0.62	0.76±0.57	0.64±0.68	0.109
treatment	PDS score	0.78±0.80	0.89±0.86	0.73±0.74	0.65±0.79	0.078	0.89±0.92	0.83±0.94	0.31±0.48	0.35±0.63	0.084	0.74±0.77	0.91±0.85	0.88±0.76	0.76±0.82	0.195
	EPS score	0.39±0.74	0.66±0.75	0.55±0.69	0.50±0.74	0.147	0.23±0.44	0.75±0.87	0.31±0.63	0.46±0.88	0.128	0.45±0.81	0.63±0.71	0.64±0.70	0.51±0.69	0.145
8-week	FD score	0.51±0.66	0.56±0.91	0.37±0.52	0.36±0.55	0.176	0.25±0.48	0.67±0.64	0.17±0.37	0.17±0.26	1.000	0.60±0.70	0.53±0.58	0.43±0.55	0.42±0.61	0.084
treatment	PDS score	0.63±0.73	0.68±0.79	0.49±0.68	0.37±0.62	0.111	0.31±0.52	0.67±0.78	0.19±0.38	0.04±0.14	0.131	0.74±0.77	0.68±0.81	0.60±0.73	0.49±0.68	0.059
	EPS score	0.39±0.76	0.44±0.63	0.24±0.47	0.34±0.58	0.094	0.19±0.48	0.67±0.62	0.15±0.38	0.31±0.43	1.000	0.46±0.83	0.37±0.62	0.27±0.49	0.35±0.62	0.219
2-week	FD score	0.61±0.67	0.58±0.65	0.29±0.53	0.29±0.60	0.614	0.73±0.40	0.67±0.72	0.23±0.56	0.06±0.21	0.686	0.56±0.74	0.55±0.63	0.30±0.52	0.36±0.67	0.357
follow-up	PDS score	0.68±0.70	0.76±0.77	0.30±0.65	0.31±0.65	0.218	0.92±0.53	0.75±0.87	0.31±0.75	0.04±0.14	0.377	0.60±0.74	0.76±0.75	0.30±0.62	0.41±0.72	1.000
	EPS score	0.53±0.79	0.40±0.67	0.27±0.49	0.26±0.59	0.060	0.54±0.52	0.58±0.67	0.15±0.38	0.77±0.28	0.746	0.53±0.87	0.34±0.67	0.31±0.52	0.32±0.66	0.219
8-week	FD score	1.99±0.77	2.04±0.94	2.06±0.97	1.63±0.62	0.757	1.73±0.62	1.98±1.09	2.02±0.83	1.48±0.62	0.140	2.07±0.81	2.05±0.90	2.07±1.02	1.68±0.62	0.557
questionnaire	PDS score	2.00±0.80	2.12±0.99	2.21±1.02	1.78±0.75	0.285	1.73±0.83	2.17±1.19	2.19±0.97	1.62±0.85	0.101	2.10±0.77	2.11±0.93	2.22±1.04	1.84±0.72	0.055
survey	EPS score	1.97±0.90	1.95±1.00	1.90±1.02	1.47±0.66	0.616	1.73±0.73	1.79±1.08	1.85±0.83	1.35±0.52	0.054	2.05±0.94	2.00±0.99	1.92±1.08	1.51±0.70	0.428

Note: [a)]FD score: the composite functional dyspepsia score is calculated as the mean of postprandial fullness, early satiety, epigastric pain, and epigastric burning scores. [b)]PDS score: the postprandial distress syndrome score is calculated as the mean of postprandial fullness score and early satiety score. [c)]EPS score: the epigastric pain syndrome score is calculated as the mean of epigastric pain score and epigastric burning score.

All hypothesis tests were two-sided. $p < 0.05$ was considered significant.

BL-99, *Bifidobacterium animalis* subsp. *lactis* BL-99.

Patients in the placebo, positive_control, BL-99_low, and BL-99_high groups were administered with maltodextrin (2 g/day), rabeprazole (10 mg/ day), low-dose BL-99 (1×10^{10} CFU/day), and high-dose BL-99 (5×10^{10} CFU/day), respectively. Source data are provided as a Source Data file.

Supplementary Table 8 Symptom score for men and women participants based on Per-Protocol (PP) Set

Symptom score		Men and women participants					Men participants					Women participants				
		Placebo (n=45)	Positive_control (n=48)	BL-99 low (n=47)	BL-99 high (n=45)	p overall	Placebo (n=13)	Positive_control (n=10)	BL-99 low (n=13)	BL-99 high (n=12)	p overall	Placebo (n=32)	Positive_control (n=38)	BL-99 low (n=34)	BL-99 high (n=33)	p overall
Baseline	FD score [a)]	1.60±0.83	1.63±0.62	1.62±0.77	1.88±0.70	0.087	1.90±0.95	2.08±0.66	1.50±0.83	2.00±0.76	0.125	1.48±0.75	1.51±0.56	1.66±0.75	1.83±0.68	0.050
	PDS score [b)]	1.93±0.75	1.97±0.45	1.95±0.77	2.11±0.72	0.234	2.11±0.77	2.15±0.58	1.69±0.78	2.17±0.75	0.157	1.86±0.74	1.92±0.41	2.04±0.76	2.09±0.72	0.196
	EPS score [c)]	1.27±1.07	1.28±0.97	1.29±0.95	1.64±0.87	0.072	1.69±1.25	2.00±0.78	1.31±0.97	1.83±0.86	0.129	1.09±0.96	1.09±0.93	1.28±0.96	1.58±0.86	0.189
4-week treatment	FD score	0.50±0.55	0.78±0.65	0.60±0.56	0.49±0.64	0.165	0.56±0.61	0.80±0.79	0.31±0.43	0.38±0.74	0.097	0.47±0.54	0.77±0.62	0.71±0.94	0.53±0.61	0.053
	PDS score	0.70±0.76	0.89±0.86	0.69±0.73	0.56±0.75	0.176	0.89±0.92	0.80±0.92	0.31±0.48	0.33±0.65	0.096	0.63±0.70	0.91±0.85	0.84±0.77	0.64±0.77	0.175
	EPS score	0.30±0.53	0.67±0.75	0.51±0.68	0.42±0.70	0.092	0.23±0.44	0.80±0.92	0.31±0.63	0.42±0.90	0.090	0.31±0.56	0.63±0.71	0.59±0.69	0.42±0.63	0.067
8-week treatment	FD score	0.41±0.52	0.55±0.58	0.31±0.46	0.24±0.41	0.110	0.25±0.48	0.65±0.61	0.17±0.37	0.13±0.20	1.000	0.48±0.53	0.53±0.58	0.36±0.48	0.29±0.46	0.084
	PDS score	0.53±0.66	0.67±0.78	0.44±0.66	0.24±0.47	0.210	0.31±0.52	0.60±0.70	0.19±0.38	0.00±0.00	0.131	0.63±0.70	0.68±0.81	0.53±0.72	0.33±0.53	0.059
	EPS score	0.30±0.56	0.44±0.63	0.18±0.38	0.24±0.47	0.142	0.19±0.48	0.70±0.62	0.15±0.38	0.25±0.40	1.000	0.33±0.59	0.37±0.62	0.19±0.39	0.24±0.50	0.219
2-week follow-up	FD score	0.52±0.54	0.57±0.64	0.22±0.46	0.17±0.46	0.839	0.73±0.40	0.65±0.71	0.23±0.56	0.00±0.00	0.686	0.43±0.57	0.55±0.63	0.22±0.43	0.23±0.53	0.357
	PDS score	0.60±0.62	0.75±0.76	0.23±0.60	0.18±0.49	0.564	0.92±0.53	0.70±0.82	0.31±0.75	0.00±0.00	0.377	0.45±0.61	0.76±0.75	0.21±0.54	0.24±0.56	1.000
	EPS score	0.44±0.62	0.40±0.68	0.21±0.41	0.16±0.48	0.055	0.54±0.52	0.60±0.70	0.15±0.38	0.00±0.00	0.746	0.41±0.67	0.34±0.67	0.24±0.43	0.21±0.55	0.219
8-week questionnaire survey	FD score	2.06±0.78	2.17±0.96	2.27±1.01	1.81±0.76	0.696	1.73±0.62	2.22±0.96	2.02±0.83	1.54±0.61	0.140	2.18±0.71	2.05±0.90	2.14±1.02	1.70±0.62	0.526
	PDS score	2.04±0.78	2.01±0.97	1.95±1.02	1.50±0.65	0.271	1.73±0.83	2.40±1.08	2.19±0.97	1.71±0.81	0.101	2.19±0.73	2.11±0.93	2.29±1.04	1.85±0.74	0.055
	EPS score	2.05±0.71	2.09±0.90	2.11±0.97	1.66±0.62	0.718	1.73±0.73	2.05±0.96	1.85±0.83	1.34±0.53	0.054	2.17±0.78	2.00±0.99	1.96±1.09	1.55±0.69	0.428

Note: [a)]FD score: the composite functional dyspepsia score is calculated as the mean of postprandial fullness, early satiety, epigastric pain, and epigastric burning scores. [b)]PDS score: the postprandial distress syndrome score is calculated as the mean of postprandial fullness score and early satiety score. [c)]EPS score: the epigastric pain syndrome score is calculated as the mean of epigastric pain score and epigastric burning score.

All hypothesis tests were two-sided. $p < 0.05$ was considered significant.

BL-99, *Bifidobacterium animalis* subsp. *lactis* BL-99.

Patients in the placebo, positive_control, BL-99_low, and BL-99_high groups were administered with maltodextrin (2 g/day), rabeprazole (10 mg/ day), low-dose BL-99 (1×10^{10} CFU/day), and high-dose BL-99 (5×10^{10} CFU/day), respectively. Source data are provided as a Source Data file.

Supplementary Table 9 Adverse events

Group		Placebo (n = 50)	Positive_control (n = 50)	BL-99_low (n = 50)	BL-99_high (n = 50)
**Number of patients with adverse events**		2 (4.0%)	1 (2.0%)	1 (2.0%)	1 (2.0%)
Digestive system	Bloating			1 (2.0%) †)	
	Nausea				1 (2.0%) †)
	Diarrhea	1 (2.0%) †)			
Skin and subcutaneous tissue	Pruritus	1 (2.0%) *‡)			
Other	Cardiac disorders		1 (2.0%) *‡)		

Data are n (%) for the full analysis set. *) Unlikely to be related to study product. †)Possibly related to study product; All adverse events in the first 8 weeks were mild (grade 1) or moderate (grade 2); ‡) Denotes adverse events leading to discontinuation.

Supplementary Table 10 Gene information related to the adhesion function of probiotics

Gene ID	Gene Name	Start	End	Length (bp)	Putative function(s)	Reference
gene0159	cpaF	186340	187407	1068	pilus assembly protein CpaF	[1]
gene0160	tadB	187407	188063	657	tight adherence protein B	
gene1459	-	1589152	1587971	1182	UPF0755 protein	
gene2119	-	2286217	2285573	645	NlpC/P60 family protein	
gene1198	talA	1339670	1338567	1104	Transaldolase	[2]
gene0786	groEL	846204	847829	1626	Chaperonin GroEL	
gene0568	-	601817	602923	1107	Outer membrane-specific lipoprotein transporter s ubunit LolE	
gene0066	-	77891	79129	1239	Sortase E	
gene0110	srtA	126202	127179	978	Sortase C	[3]
gene0168	-	196674	195541	1134	Sortase C	
gene1631	-	1771060	1772007	948	Sortase C	

2. Supplementary Figures

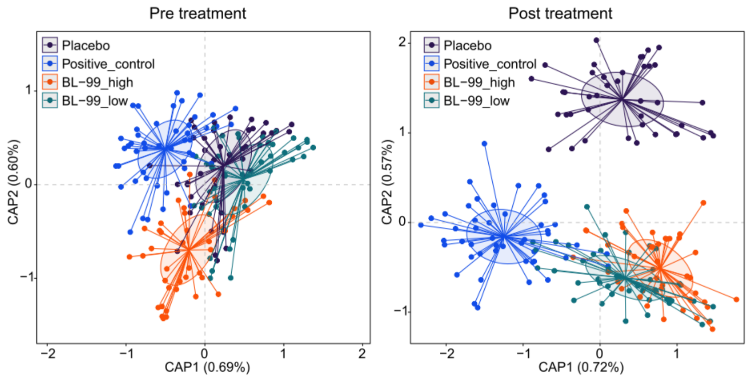

Supplementary Fig. 1. Distance-based redundancy analysis (dbRDA) of microbiota communities in the fecal samples among four groups at baseline and post treatment period. Samples are shown at the first and second principal coordinates (CAP1 and CAP2), and the ratio of variance contributed by these two CAPs is shown. Ellipsoids represent a 95% confidence interval surrounding each group. The below and left boxplots show the sample scores in CAP1 and CAP2 (boxes show medians/quartiles; error bars extend to the most extreme values within 1.5 interquartile ranges). Patients in the placebo (n = 45), positive_control (n = 48), BL-99_low (n = 47), and BL-99_high (n = 45) groups were administered with maltodextrin (2 g/day), rabeprazole (10 mg/day), low-dose BL-99 (1×10^{10} CFU/day), and high-dose BL-99 (5×10^{10} CFU/day), respectively.

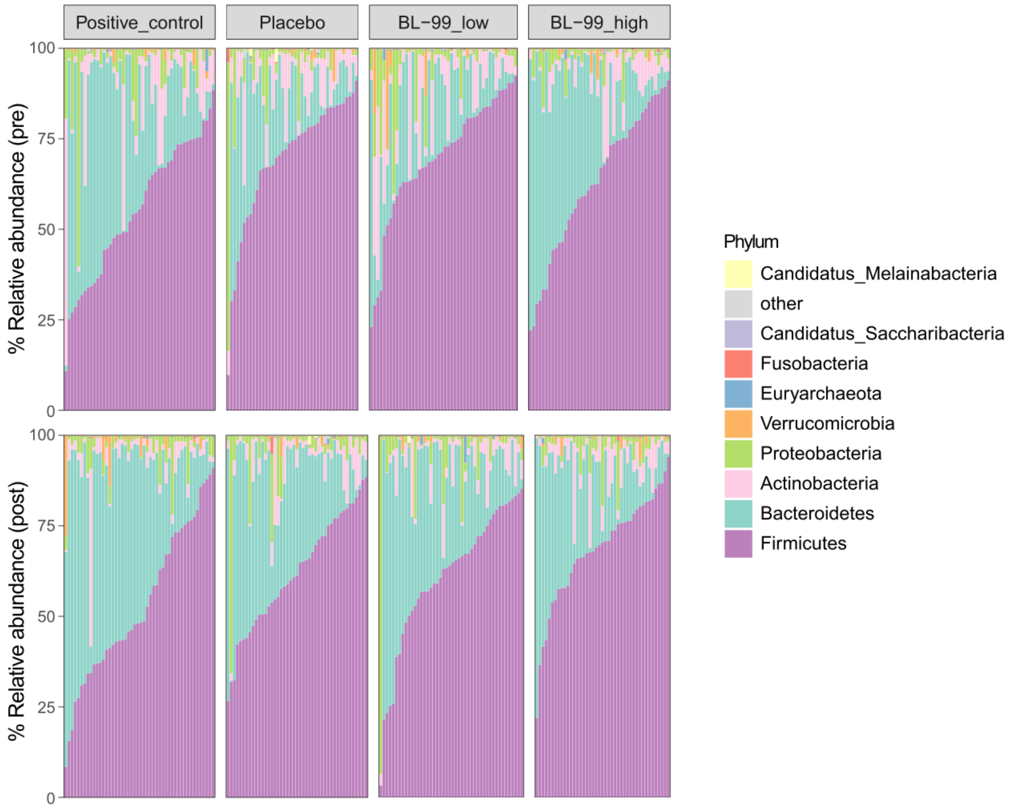

Supplementary Fig. 2. Composition of the gut microbiota of samples at baseline and post treat period.

Data are shown at the phylum level. Patients in the placebo (n = 45), positive_control (n = 48), BL-99_low (n = 47), and BL-99_high (n = 45) groups were administered with maltodextrin (2 g/day), rabeprazole (10 mg/day), low-dose BL-99 (1×10^{10} CFU/day), and high-dose BL-99 (5×10^{10} CFU/day), respectively.

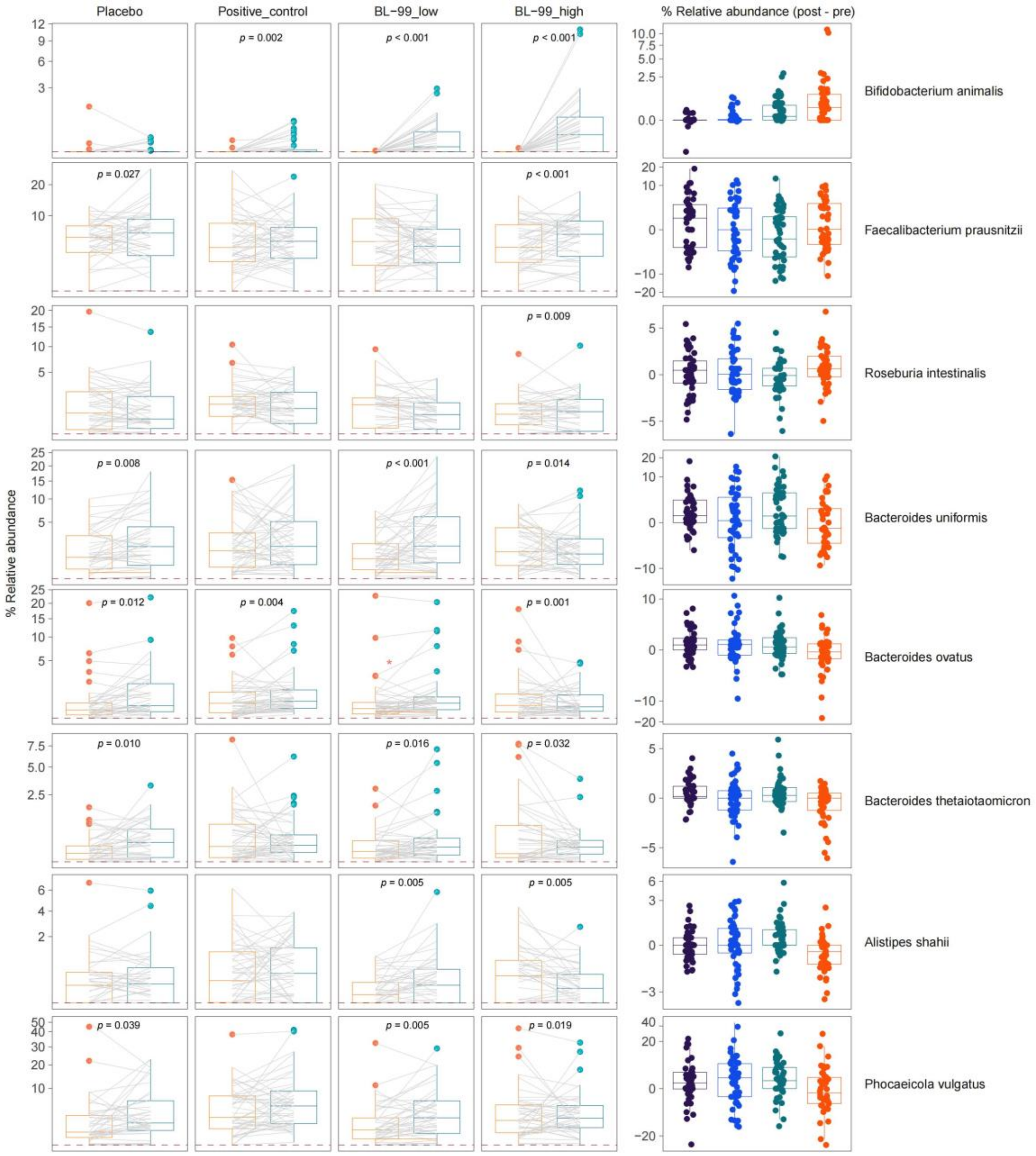

Supplementary Fig. 3. Changes of species from the baseline to the post treatment period. For each species, the left four panels show the changes in relative abundances between the baseline and post treatment period in four groups.

Boxes represent the interquartile range between the first and third quartiles and the median (internal line). Whiskers denote the lowest and highest values within 1.5 times the range of the first and third quartiles, respectively; dots represent outlier samples beyond the whiskers. *p*-values are calculated using the two-side Wilcoxon rank-sum test. For each species, the right panel shows the changes in relative abundance of species from pre to post treatment for samples. Patients in the placebo (n = 45), positive_control (n = 48), BL-99_low (n = 47), and BL-99_high (n = 45) groups were administered with maltodextrin (2 g/day), rabeprazole (10 mg/day), low-dose BL-99 (1×10^{10} CFU/day), and high-dose BL-99 (5×10^{10} CFU/day), respectively.

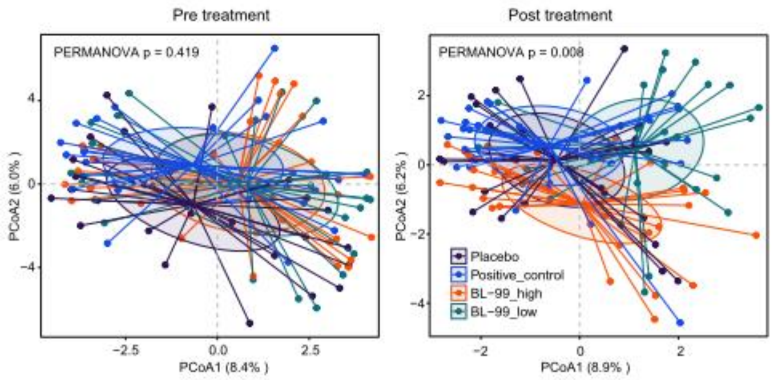

Supplementary Fig. 4. Principal coordinates analysis (PCoA) of fecal metabolome among four groups at baseline and post treatment period.

Samples are shown at the first and second principal coordinates (PCoA1 and PCoA2), and the ratio of variance contributed by these two PCoAs is shown. Ellipsoids represent a 95% confidence interval surrounding each group. The below and left boxplots show the sample scores in PCoA1 and PCoA2 (boxes show medians/quartiles; error bars extend to the most extreme values within 1.5 interquartile ranges). Patients in the placebo (n = 45), positive_control (n = 48), BL-99_low (n = 47), and BL-99_high (n = 45) groups were administered with maltodextrin (2 g/day), rabeprazole (10 mg/day), low-dose BL-99 (1×10^{10} CFU/day), and high-dose BL-99 (5×10^{10} CFU/day), respectively.

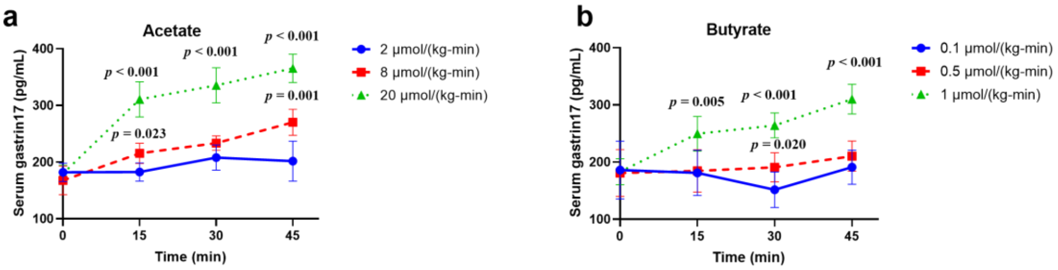

Supplementary Fig. 5. Effect of short-chain fatty acid infusion on serum gastrin.
a acetate. **b** butyrate. Significant differences among different groups were evaluated by one-way analysis of variance (ANOVA) with least significant difference (LSD) analysis *vs*. 2 μmol/(kg-min) acetate or 0.1 μmol/(kg-min) butyrate). All hypothesis tests were two-sided. $p < 0.05$ was considered significant. Source data are provided as a Source Data file.

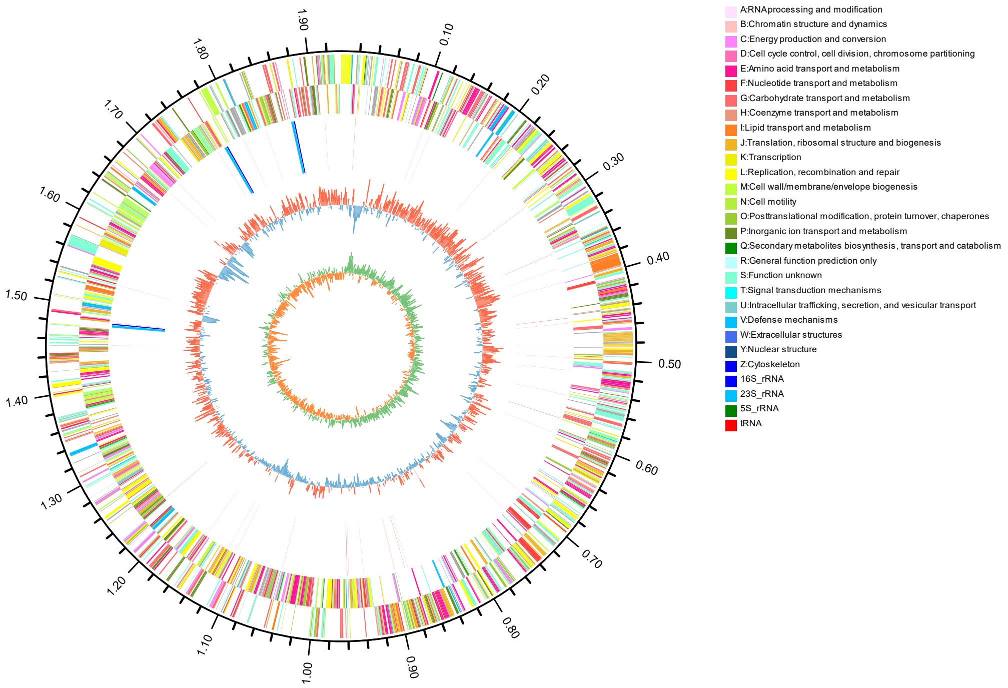

Supplementary Fig. 6 The 16s gene map of Bifidobacterium animalis subsp. lactis. BL-99. The outermost circle is the identification of the size of the genome; The second and third circles are CDS on the positive and negative chains, and different colors indicate the functional classification of different COGs of CDS; The fourth circle is rRNA and tRNA; The fifth circle is the GC content, the outward red part indicates that the GC content in this region is higher than the whole genome average GC content, and the inward blue part indicates that the GC content in this region is lower than the whole genome average GC content. The innermost circle is the GC-Skew value.

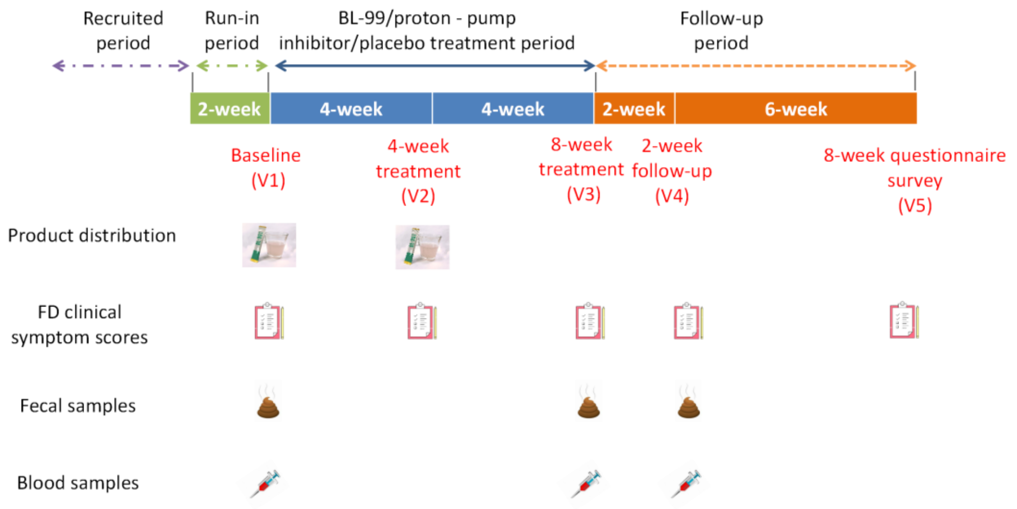

Supplementary Fig. 7. The study design of the trial

3. Supplementary Methods

3.1 Metagenomic Analysis

Of the 185 participants who completed the entire trial, 94.6% had complete fecal samples and were used for whole-metagenome shotgun sequencing based on the lumina NovaSeq PE150 (Illumina Inc., San Diego, CA, USA) platform at Majorbio Bio-Pharm Technology Co., Ltd. (Shanghai, China). Metagenomic sequencing was carried out based on the methods as previously described[4]. In brief, total genomic DNA was extracted from human feces using the E.Z.N.A.® Soil DNA Kit (Omega Bio-tek, Norcross, GA, U.S.) according to manufacturer's instructions. And all the concentration and purity of total genomic DNA were determined by TBS-380 micro fluorometer (Turner Bio Systems, USA) and NanoDrop2000 ultra-micro spectrophotometer (Thermo Fisher Scientific, USA) respectively. The raw sequencing reads were processed for quality controls using fastp (0.23.0)[5]. Low quality (>45 bases with quality score <20, or >5 'N' bases), low complexity, and adapter-containing reads were removed, and the remaining reads were trimmed at the tails for low quality (<Q20) or 'N' bases. Human genomic reads were removed via mapping to the reference human genome (GRCh38) using Bowtie2 (2.4.4)[6]. The gut microbiota were then compositionally quantified using the MetaPhlAn4 (4.0.2) algorithms[7]. Analysis of biosynthesis capacity of short-chain fatty acids (SCFAs), biles, and uremic toxins was realized following our previous method[8]. Briefly, we used the presence of key synthetases to denote the biosynthesis capacity of such molecules for each MAG: acetate synthase (acetyl-CoA decarbonylase/synthase), propionate synthase I (lactoyl-CoA dehydratase), propionate synthase II (propionaldehyde dehydrogenase), butyrate synthase I (butyryl-CoA:acetate CoA-transferase), butyrate synthase II (butyrate kinase), bile salt hydrolase, 7α/β-dehydroxylation enzymes, hydroxysteroid dehydrogenase, tryptophanase, tyrosine phenol-lyase, 4-hydroxyphenylacetate decarboxylase (*hpdC*), phenyllactate dehydrogenase/dehydratase (*fldHBC*), pyruvate:ferredoxin oxidoreductase A (*proA*), and choline trimethylamine-lyase.

3.2 Non-target metabolic

Fecal metabolites extration and analysis followed a published method with modification[9]. Brifely, 200 mg fecal sample, 1.5 g sodium chloride, 3 mL distilled water and 20 μL internal standard solution (7.55 mg/L 2-methyl-3-heptanone) were added to a 20 mL headspace vail, and then vortexed well. Quilibration for 15 min at 65°C, the solid-phase microextraction (SPME) fiber (DVB/CAR/PDMS, 50/30 μm, Supelco, Bellefonte, PA, USA) was placed in the headspace for 30 min. Then, the SPME fiber with the analytes was adsorbed and analysed by Agilent gas chromatography quadrupole-time-of-flight mass spectrometer (GC-Q-TOF, 7200-7890B, Agilent Thechnologies, Santa Clara, CA, USA) with Agilent DB-WAX capillary column (30 m × 250 μm × 0.25 μm). The column temperature for the detection of the fecal sample was initially set at 40°C for 1 min, increased by 8°C/min to 120°C, then increased by 5°C/min until reaching 230°C, held for 10 min, with the total time of 43 min. The other conditions of MS were set as follows: the carrier gas was helium gas at a constant flow rate of 1 mL/min, the collision voltage was 70 eV, and the ion source temperature was set at 230°C. The mass spectrum scan range was 40–400 *m/z*.

The data sets were processed for peak pick and deconvolution with Unknowns Analysis tool of the MassHunter Quantitative Analysis software package (B.10.1, Agilent Technologies). Mass Profiler Professional Software (MPP) (version 14.5, Agilent Technologies) was used for alignment, normalization and annotation. The mass spectra of volatile substances were matched to the

reference mass spectra in the NIST17 library, and those with a matching factor above 75 were selected for data processing. Alignment was based on the *m/z* and retention time, and quantitation normalization was based on the peak area of the internal standard.

3.3 Short Chain Fatty Acids (SCFAs) Measures

Short chain fatty acids (SCFAs) were detected using the gas chromatography method as described in previous study[10]. Briefly, 50 μL 50% sulfuric acid was added to the 20 mg fecal sample/ 20 μL serum, and the homogenizer was used to homogenize the sample for 3 min at 4°C (6 cycle; 30 s/c; pause for 10 s). Then 10 μL internal standard (2.927 mmol/L 2-ethylbutyric acid) and 500 μL ether were added and homogenized for 1 min (2 cycle; 30 s/c; pause for 5 s). Centrifugation was performed at 4600 x g for 5 min at 4°C, and 350 μL supernatant was taken. After that, 350 μL supernatant were taken by centrifuging at 4600 x g for 5 min (4°C). 500μL ether was added to the precipitation solution, and 400 μL supernatant were taken after homogenizing and centrifuging. Finally, the mixed supernatant was filtered by 0.22 μm filter membrane, and injected into a brown sample bottle for Agilent GC-8860 gas chromatograph analysis.

Chromatographic conditions: HP-FFAP column (30 m * 250 μm * 0.25 μm, Agilent Technologies, Inc.); Carrier gas: high-purity nitrogen; Column flow rate: 1 mL/min; Temperature of FID detector: 250°C, H_2, air and tail air flow were set as 30, 400 and 25 mL/min respectively. The injection volume was 1 μL, and the temperature program was as follows:

$$60°C\ (4\ min) \xrightarrow{6°C/min} 180°C\ (0\ min) \xrightarrow{20°C/min} 200°C\ (5\ min)$$

3.4 SCFA infusion experiment

3.4.1 Ethics statement

All the experimental procedures were approved by the Ethics Committee of Beijing Laboratory Animal Research Center (approval No. BLARC-LAWER-202306006), and were also in accordance with the Guidelines for the Care and Use of Laboratory Animals published by the US National Institutes of Health.

3.4.2 Animals

Thirty-six normal male Sprague-Dawley rats (8-9 weeks of age, 350-390 g) were ordered from Charles River Laboratories (CRL, Beijing, China). All animals were housed in the SPF animal housing facility with a 12-h on/12-h off light cycle, 20-26 ℃ ambient temperature, 40-70% humidity, and free access to food (batch No. 0515SH05190325C) and water. And all animals were allowed to acclimate for 7 d, fasted for 12 h (overnight), and randomly divided into 6 groups (n=6) prior to SCFA infusion experiments.

3.4.3 Intracarotid SCFA infusion experiments

The intracarotid SCFA infusion experiments were performed following previously described protocol[11], with some modification. Rats were general anesthetized with isoflurane (2% induction, 2% maintenance in 70% N_2 and 30% O_2). A femoral artery catheter was used to monitor arterial blood pressure. Rectal temperature was monitored and maintained at 37 °C via a servo-controlled heating pad. PE50 tubing was inserted retrogradely into the external carotid artery and advanced into the carotid bifurcation. And then 2, 8, 20 μmol/(kg-min) acetate and 0.1, 0.5, 1 μmol/(kg-min)

butyrate were infused immediately for 45 min, and the animals were euthanized. Blood samples (200 μL) were collected during the infusion period (0, 15, 30, and 45 min). Serum sample was supernatants obtain from blood samples after centrifugation at 1006.2 x g for 15 min. And serum gastrin was measured using mlbio ELISA kit (Shanghai Enzyme-linked Biotechnology Co., Ltd, China)

3.4.4 Statistical analysis

Serum gastrin levels were described as mean (M) ± standard deviation (SD). And significant differences among different groups were evaluated by one-way analysis of variance (ANOVA) with least significant difference (LSD) analysis. All hypothesis tests were two-sided. $p < 0.05$ was considered significant.

4. Supplementary Note1: FD symptom questionnaire

Symptom Questionnaire[12,13]

No. ____________________ Date:______________________

Date of Birth:_______________ Gender:____________________

Height:____________________ Weight:____________________

Clinical symptom	Clinical symptom scores
1. Have you had postprandial fullness for 6 months or longer?	0 No<br>1 Yes
a. Usually how severe was the postprandial fullness	0 None<br>1 Mild<br>2 Moderate<br>3 Severe
2. Have you had early satiety for 6 months or longer?	0 No<br>1 Yes
a. Usually how severe was this feeling?	0 None<br>1 Mild<br>2 Moderate<br>3 Severe
3. Have you had epigastric pain or discomfort for 6 months or longer?	0 No<br>1 Yes
a. Usually how severe was this feeling?	0 None<br>1 Mild<br>2 Moderate<br>3 Severe
4. Have you had epigastric burning for 6 months or longer?	0 No<br>1 Yes
a. Usually how severe was the epigastric burning?	0 None<br>1 Mild<br>2 Moderate<br>3 Severe
5. Have you had bothersome nausea for 6 months or longer?	0 No

	1 Yes
a. Usually how severe was the nausea?	0 None<br>1 Mild<br>2 Moderate<br>3 Severe
6. Did you experience bothersome belching more than 6 months ago?	0 No<br>1 Yes
a. Do you have belching before meals?	0 No<br>1 Yes
b. Do you have belching during meals	0 No<br>1 Yes
c. Do you have belching after meals?	0 No<br>1 Yes
d. Do you have belching unrelated to meals?	0 No<br>1 Yes
e. Usually how severe was the belching?	0 None<br>1 Mild<br>2 Moderate<br>3 Severe
7. Have you had acid reguritation for 6 months or longer?	0 No<br>1 Yes
a. Is this uncomfortable fullness related to meal?	0 No<br>1 Yes, after a large meal<br>2 Yes, after a regular meal<br>3 Yes, after a small meal
b. How do you define the severity of your fullness?	0 None<br>1 Mild<br>2 Moderate<br>3 Severe
8. Have you had vomiting for 6 months or longer?	0 No<br>1 Yes

a. Did you make yourself vomit?		0 Never<br>1 Sometimes<br>2 Often<br>3 Always
9. What is your average stool frequency per week?		________ no./week
10. Indicate the type of stool you usually pass		BSS no.__________
Bristol stool Scale Form (BSS)		
Type 1	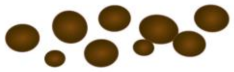	Separate hard lumps like nuts (hard to pass)
Type 2	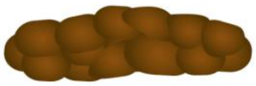	Sausage-shaped but lumpy
Type 3	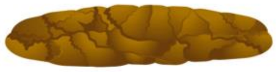	Like a sausage but with cracks on its surface
Type 4	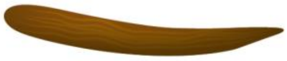	Like a sausage or snake, smooth and soft
Type 5	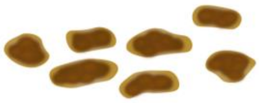	Soft blobs with clear-cut edges (passed easily)
Type 6	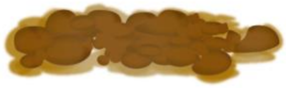	Fluffy pieces with ragged edges, a mushy stool
Type 7	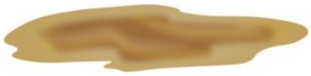	Watery, no solid pieces (entirely liquid)

5. Supplementary Note2: Study protocol

Clinical study on *Bifidobacterium* BL-99 assisting to improve functional dyspepsia

Qi Zhang (809425044@qq.com)

Department of Nutrition and Health, Key Laboratory of Functional Dairy, Co-constructed by Ministry of Education and Beijing Government, China Agricultural University, Beijing, China; https://orcid.org/0000-0002-9021-3960

Fazheng Ren (renfazheng@263.net)

Department of Nutrition and Health, Key Laboratory of Functional Dairy, Co-constructed by Ministry of Education and Beijing Government, China Agricultural University, Beijing, China

Keywords: Functional dyspepsia, *Bifidobacterium animalis* subsp. *lactis* BL-99, efficacy, gastrointestinal microbiota

Abstract

Functional dyspepsia (FD) is a common chronic gastrointestinal disorder without known organic pathology changes. Recurrence symptoms and longer duration resulting in poor quality of life and high medical expenses for patients. Therefore, searching for a prolonged treatment of FD has considerable clinical value. There are two objectives in this research, one is to determine efficacy of BL-99 to improve FD symptoms and the underlying mechanism, and the other is to evaluate the impact of BL-99 on gastrointestinal microbiota after proton pump inhibitors (PPI) therapy. In this randomized controlled trial, corresponding to the research purpose 1, subjects will be randomized into four groups (mild placebo group, mild low dose group, mild high dose group, severe placebo group), and corresponding to the research purpose 2, subjects will be randomized into two groups (severe low dose group, and severe high dose group). Except the mild placebo group, other groups will receive BL-99 and/or PPI. The clinical trial registry is at Chictr.org.cn ID ChiCTR2000041430.

Introduction

Functional dyspepsia (FD) is a common chronic gastrointestinal disorder without known organic pathology changes[14]. FD is subdivided into Epigastric pain syndrome (EPS, including epigastric pain or epigastric burning symptoms) and Postprandial distress syndrome (PDS, including bloating or early satiety symptoms]). Currently, there is a lack of efficient treatment for this refractory disease[15], causing poor life quality and higher medical expenses for patients[16]. Therefore, searching for a prolonged treatment of FD has considerable clinical value.

Several anti-dyspepsia drugs (acid-suppressive therapy, prokinetics, neuro-modulators and herbal therapies) have been recommended to treat FD symptoms in clinical practice, with significant side effects and unknown long-term efficacy[17]. Among them, PPI are considered to be the most effective and first-line therapy but its long-term effect is limited, which may be related to the change of gut microbiota and the increased risk of intestinal infection[18]. Hence, it is an urgent need to develop an efficient, targeted and long-term therapy for FD.

Probiotics have been shown to have the potential to alleviate FD. Consuming probiotic products containing *Lactobacillus gasseri* OLL2716, *Lactobacillus paracasei* LC-37, et al., can significantly improve postprandial discomfort, epigastric pain, belching and other FD symptoms[19-21]. More importantly, Khoder G et al.[22] demonstrated that probiotics could regulate the release of serum pepsinogen. However, the efficacy of different strains varies, and the mechanism of probiotic intervention to alleviate FD needs to be further studied.

Bifidobacterium animalis subsp. *lactis*. BL-99 (BL-99) was isolated from the faeces of healthy infant. *In vitro* and *in vivo* experiments showed that BL-99 is a non-pathogenic strain and could be used in the production of functional food[23]. Then, it was confirmed that BL-99 could maintain intestinal health by reducing intestinal inflammation[24]. Moreover, recent studies suggested that BL-99 could utilize fructo-oligosaccharide (FOS) to regulate the composition and structure of gut microbiota in patients with constipation, thus promoting the production of short chain fatty acids (SCFAs)[25]. Hence, a randomized parallel controlled trial will be performed

to study the prolonged efficacy of BL-99 to improve FD symptoms and the underlying mechanism, and evaluate the impact of BL-99 on gastrointestinal microbiota after PPI therapy.

Reagents and equipment

NA

Procedure

Sample Size Calculation:

Based on previous studies, the power for the primary outcome reached 80% (two-sided test, $\alpha = 0.05$) with a sample size of 42 participants per group[26]. This study assumed that the overall dropout rate would be 10-12% during the entire study period. To account for probable follow-up loss, 50 study participants per group were recruited.

Inclusion Criteria:

(1) Aged 18-60 years;

(2) Meet one or more symptoms of Rome IV FD diagnostic criteria (epigastric pain, belching, early satiety, epigastric burning, acid regurgitation, bloating, nausea and vomiting), the course of the disease is more than 6 months, and symptoms have occurred in the past three months;

(3) Willing to provide demographic information (age, weight, height, etc.);

(4) Be willing to and complete daily and weekly questionnaires on general health, intestinal function and gastrointestinal symptoms.

Exclusion Criteria:

(1) Indigestion caused by serious organic lesions (cardiovascular, liver, kidney, or hematopoietic) diseases and without *Helicobacter pylori* (*H. pylori*) infection (diagnosed by the C^{14}-urea breath test);

(2) Pregnancy;

(3) Smokers and alcoholics;

(4) Take any medication for constipation or diarrhea;

(5) Currently taking any anti-inflammatory medication regularly;

(6) Usually eat fermented foods or probiotics (for example, kimchi, sauerkraut, fermented bean curd, tempeh, cheese);

(7) Received antibiotics in the past three months;

(8) A history of clinically significant diarrhea or C. diffinfection in the past 3 months.

Group and intervention:

Group and inervention corresponding to the research purpose 1:

(1) Mild placebo group: the placebo contained 2 g/day maltodextrin.

(2) Mild low dose group: 2 g solid beverages containing 1×10^{10} CFU/day BL-99.

(3) Mild high dose group: 2 g solid beverages containing 5×10^{10} CFU/day BL-99.

(4) Severe placebo group: 10 mg/day proton pump inhibitors (PPI).

Group and intervention corresponding to the research purpose 2:

(5) Severe low dose group: 2 g solid beverages containing 1×10^{10} CFU/day BL-99 and 10 mg/day PPI.

(6) Severe high dose group: 2 g solid beverages containing 5×10^{10} CFU/day BL-99 and 10 mg/day PPI.

Time Taken

After a 2-week run-in period, the participants underwent an 8-week intervention period; and an 8-week follow-up period.

Anticipated results

The prolonged efficacy of BL-99 to improve FD symptoms and the underlying

mechanism will be explored. Moreover, the impact of BL-99 on gastrointestinal microbiota after PPI therapy will be evaluated.

Supplementary References

6. Supplementary Note3: Statistical analysis plan

Statistical analysis plan for the "Clinical study on *Bifidobacterium* BL-99 assisting to improve functional dyspepsia"

This study includes 2 sub-studies, which correspond to different research purposes. Therefore, the following statistical analysis plans are presented separately according to sub-studies.

Sub-study 1

1. Research purpose

(1) Main purpose

To evaluate the effect of *Bifidobacterium animalis* subsp. *lactis* BL-99 （BL-99） probiotics intervention on symptoms of functional dyspepsia (FD) in FD population.

(2) Secondary purpose

To study the effect of BL-99 probiotics intervention on gut microbiota and metabolites of FD population.

2. Research design

2.1 Overall design

This study is a randomized, open, parallel controlled study involving 4 intervention groups.

Randomization method: A computer-generated list of random numbers is used to randomly assign the participants to the 4 groups.

2.2 Group and Intervention

**Intervention groups**	**Intervention substances**	**Intervention time**
(1) Mild placebo group	2 g/day maltodextrin	8 weeks
(2) Mild low dose group	2 g solid beverages containing 1 × 10^{10} CFU/day BL-99	8 weeks
(3) Mild high dose group	2 g solid beverages containing 5 × 10^{10} CFU/day BL-99	8 weeks
(4) Severe placebo group	10 mg/day proton pump inhibitors	8 weeks

	(PPI)	

2.3 Time Taken

After a 2-week run-in period, the participants underwent an 8-week intervention period; and an 8-week follow-up period. There will be two intervention visits, one at 4 weeks of intervention and one at 8 weeks of intervention. After the intervention, in order to evaluate the long-term effect of probiotic intervention on the improvement of dyspepsia symptoms, follow-up will be continued for 8 weeks, and 2 visits will be set at 2 weeks and 8 weeks post the intervention, respectively.

3. Outcome indicators

3.1 Main outcome indicators

Clinical response rate of PDS+EPS score at 8 weeks of intervention. Clinical response rate was defined as a score reduction of ≥0.5 from pre-intervention. (PDS: postprandial distress syndrome; EPS: epigastric pain syndrome)

3.2 Secondary outcome indicators

- Clinical response rate of PDS+EPS at 4 weeks of intervention, 2 weeks after the intervention, and 8 weeks after the intervention.
- Clinical response rate of PDS and EPS scores at 4 weeks of intervention, at 8 weeks of intervention, 2 weeks after the intervention, and 8 weeks after the intervention.
- Changes of serum index values reflecting gastric digestion ability at 8 weeks of intervention, and 2 weeks after the intervention, mainly including serum pepsinogen I (PG I), pepsinogen II (PG II), pepsinogen ratio (PGR) = PG I/PG II, and gastrin 17 (G17).
- Gut microbiota at 8 weeks of intervention.
- Faecal metabolites at 8 weeks of intervention.
- Fecal and Serum Short chain Fatty Acids (SCFA) at 8 weeks of intervention.

3.3 Safety indicators

Adverse event rate: Participants are asked to report any adverse effects during the treatment and follow-up periods, such as bloating, nausea, diarrhea, itchy skin, etc. Safety is assessed by classifying adverse events using the Common Terminology Criteria for Adverse Events (CTCAE) version 5.0 at each study period or in the case

of early termination.

4. Sample size calculation

Based on previous studies, the power for the primary outcome reached 80% (two-sided test, $\alpha = 0.05$) with a sample size of 42 participants per group. This study assumes the overall dropout rate to be 10-12% during the entire study period. To account for probable follow-up loss, at least 200 participants will be recruited.

5. Data sets of statistical analysis

(1) Per-Protocol (PP) Set

The main data set for efficacy analysis in this study is PP. PP refers to all participants that have completed the planned intervention and visits according to the protocol and have no obvious effect on the therapeutic effect. Violations that significantly affect efficacy are determined at the time of data review and may include (but are not limited to) the following: ① failure to meet inclusion criteria; ② there was interference therapy after inclusion; ③ poor compliance; ④ follow-up beyond the window period.

(2) Safety Set (SS)

The main data set for safety analysis in this study is SS. Safety evaluation data of participants in 8-week intervention and 8-week follow-up period constituted the SS of this study.

6. Statistical analysis

6.1 Software

Statistical analysis of clinical indicators will be performed using SPSS Statistics 24 (SPSS Institute, Chicago, IL, USA). Figures other than those related to microbial analysis will be created using GraphPad Prism 9.0.0. Fecal metabolites will be processed for peak pick and deconvolution with Unknowns Analysis tool of the MassHunter Quantitative Analysis software package (B.10.1, Agilent Technologies). Mass Profiler Professional Software (MPP) (version 14.5, Agilent Technologies) will be used for alignment, normalization and annotation. Metagenomics analysis will be performed using the online platform of Majorbio Cloud Platform (www.majorbio.com). And fastp v0.20.0 (https://github.com/OpenGene/fastp),

MEGAHIT v1.1.2 (https://github.com/voutcn/megahit) and MetaGene (http://metagene.cb.k.u-tokyo.ac.jp/) software will be used for statistics and quality control of raw sequencing data, assembly of sequencing data and gene prediction. NR (nr_20200604, https://ftp.ncbi.nlm.nih.gov/blast/db/FASTA/), eggnog (v4.5.1, http://eggnog5.embl.de/#/app/downloads), KEGG (v94.2, https://www.genome.jp/kegg) and CAZy (v8, http://bcb.unl.edu/dbCAN2/download/Data) will be used for species, COG, KEGG, and CAZy annotation.

6.2 General principles

All hypothesis tests are two-sided. In general, $P < 0.05$ is considered significant, except for multiple comparisons, where the threshold of significance is 0.008. In the analysis of intestinal microorganisms and metabolites, the level of significance is further set at $P < 0.05$, *; $P < 0.01$, **; $P < 0.001$, ***; $P < 0.0001$, ****.

6.3 Subject enrollment and completion summary

A flowchart will be used to summarize the enrollment and completion of the study.

6.4 Description and comparison of baseline characteristics

Baseline demographic characteristics (age, sex, body mass index) and clinical characteristics of FD (bloating, early satiety, Epigastric pain, Epigastric burning, PDS, EPS, and Total score) will be described between groups. Continuous variables are described as mean and 95% confidence interval (95%CI). Counting variables are described as frequency and percentage.

For comparison between groups, One-way analysis of variance (ANOVA) or Kruskal-Wallis rank test is used for continuous variables, and chi-square test is used for counting variables.

6.5 Effect and safety analysis after intervention

(1) Clinical response rate of FD

Clinical response rates of PDS+EPS, PDS and EPS scores are calculated for each group at 4 weeks and 8 weeks of intervention, 2 weeks and 8 weeks of post-intervention follow-up. Chi-square test is used to compare the differences in response rates between the groups, and logistic regression is used to calculate the

relative risk (RR) and 95% confidence interval (95%CI).

In order to explore whether the effects of probiotic interventions differ between gender groups, analyses of clinical response rates will be conducted separately in the general population, in men, and in women.

(2) Serum indexes reflecting gastric digestibility

The change values of each index from baseline to 8 weeks of intervention (△1), and from 8 weeks of intervention to 2 weeks post the intervention (△2) are described respectively, and least-squares means and 95%CIs are calculated.

Comparisons of △1 and △2 between groups are performed by ANOVA. If the overall difference between the groups is significant, least significant difference (LSD) method was used for multiple comparison. The statistical significance level of P-values for multiple comparisons is set at $P<0.008$ using Bonferroni correction.

(3) Analysis of intestinal microorganisms and metabolites

ANOVA with LSD method is used to analyze the differences of α diversity indexes, the relative abundance of phyla and species, and the microbiome function among the four groups. Non-parametric test with Kruskal-Wallis is applied to detect differences in the un-target metabolites among the four groups. Paired T test is used to analyze the significance of short chain fatty acids before and after intervention. And the correlations between the relative abundance of species and short chain fatty acids are assessed by Spearman’s correlation analysis.

(4) Safety analysis

Frequency and percentage are used to describe the incidence of various adverse events in each group.

Sub-study 2

1. Research purpose

To study the improvement effect of BL-99 probiotic intervention on drug-induced gut microbiota disturbance in functional dyspepsia (FD).

2. Research design

2.1 Overall Design

This is a randomized, double-blind, parallel-controlled study involving 2 intervention groups.

Randomization: A computer-generated list of random numbers is used to randomly assign the participants to the 2 intervention groups.

Blinding: Products blinding will be achieved by preparing probiotics as solid beverages with similar packaging, smell, and taste. Independent statisticians will analyze the data without knowing the specific allocation of interventions.

2.2 Group and intervention

Intervention groups	Intervention substances	Intervention time
(5) Severe low dose group	2 g solid beverages containing 1×10^{10} CFU/day BL-99 and10 mg/day PPI	8 weeks
(6) Severe high dose group	2 g solid beverages containing 5×10^{10} CFU/day BL-99 and10 mg/day PPI	8 weeks

2.3 Time Taken

After a 2-week run-in period, the participants underwent an 8-week intervention period; and an 8-week follow-up period. There will be two intervention visits, one at 4 weeks of intervention and one at 8 weeks of intervention. After the intervention, in order to evaluate the long-term effect of probiotic intervention on the improvement of gut microbiota disturbance, follow-up will be continued for 8 weeks, and 2 visits will be set at 2 weeks and 8 weeks post the intervention, respectively.

3. Outcome indicators

3.1 Main outcome indicators

Gut microbiota at 8 weeks of intervention.

3.2 Secondary outcome indicators

- ◆ clinical response rate of PDS+EPS, PDS and EPS scores at 4 weeks of intervention, at 8 weeks of intervention, and 2 weeks after the intervention (PDS: postprandial distress syndrome; EPS: epigastric pain syndrome).

- changes of serum index values reflecting gastric digestion ability at 8 weeks of intervention, and 2 weeks after the intervention, mainly including serum pepsinogen I (PG I), pepsinogen II (PG II), pepsinogen ratio (PGR) = PG I/PG II, and gastrin 17 (G17).
- gut microbiota 2 weeks after the intervention.
- fecal metabolites at 8 weeks of intervention.

4. Sample size calculation

The main outcome of this study was the gut microbiota after intervention. There is no suitable reference for sample size calculation. Considering the consistency of the included population, the sample size of Sub-study 2 was determined in accordance with that of Sub-study 1, that is, 50 subjects were planned to be enrolled in each group.

5. Data set of statistical analysis

Per-Protocol (PP) Set

The main data set for efficacy analysis in this study is PP. PP refers to all participants that have completed the planned intervention and visits according to the protocol and have no obvious effect on the therapeutic effect. Violations that significantly affect efficacy are determined at the time of data review and may include (but are not limited to) the following: ① failure to meet inclusion criteria; ② interference therapy after inclusion; ③ poor compliance; ④ follow-up beyond the window period.

6. Statistical analysis

6.1 Software

Figures other than those related to microbial analysis will be created using GraphPad Prism 9.0.0. Fecal metabolites will be processed for peak pick and deconvolution with Unknowns Analysis tool of the MassHunter Quantitative Analysis software package (B.10.1, Agilent Technologies). Mass Profiler Professional Software (MPP) (version 14.5, Agilent Technologies) will be used for alignment, normalization and annotation. Metagenomics analysis will be performed using the online platform of Majorbio Cloud Platform (www.majorbio.com). And fastp v0.20.0

(https://github.com/OpenGene/fastp), MEGAHIT v1.1.2 (https://github.com/voutcn/megahit) and MetaGene (http://metagene.cb.k.u-tokyo.ac.jp/) software will be used for statistics and quality control of raw sequencing data, assembly of sequencing data and gene prediction. NR (nr_20200604, https://ftp.ncbi.nlm.nih.gov/blast/db/FASTA/), eggnog (v4.5.1, http://eggnog5.embl.de/#/app/downloads), KEGG (v94.2, https://www.genome.jp/kegg) and CAZy (v8, http://bcb.unl.edu/dbCAN2/download/Data) will be used for species, COG, KEGG, and CAZy annotation. Statistical analysis of clinical indicators will be performed using SPSS Statistics 24 (SPSS Institute, Chicago, IL, USA).

6.2 General principles

All hypothesis tests are two-sided. In general, $P < 0.05$ is considered significant. In the analysis of intestinal microorganisms and metabolites, the level of significance is further set at $P < 0.05$, *; $P < 0.01$, **; $P < 0.001$, ***; $P < 0.0001$, ****.

6.3 Subject enrollment and completion summary

A flowchart will be used to summarize the enrollment and completion of the study.

6.4 Description and comparison of baseline characteristics

Baseline demographic characteristics (age, sex, body mass index) and clinical characteristics of FD (bloating, early satiety, Epigastric pain, Epigastric burning, PDS, EPS, and Total score) will be described between groups. Continuous variables are described as mean and 95% confidence interval (95%CI). Counting variables are described as frequency and percentage.

For comparison between groups, independent t test or Wilcoxon rank test is used for continuous variables, and chi-square test is used for counting variables.

6.5 Effect analysis after intervention

(1) Analysis of intestinal microorganisms and metabolites

Independent t test is used to analyze the differences of α diversity indexes, the relative abundance of phyla and species, and the microbiome function between the 2 groups. Wilcoxon rank test is applied to detect differences in the un-target metabolites between the 2 groups. Paired t test is used to analyze the significance of short chain

fatty acids before and after intervention. And the correlations between the relative abundance of species and short chain fatty acids are assessed by Spearman's correlation analysis.

(2) Clinical response rate of FD

Clinical response rates of PDS+EPS, PDS and EPS scores are calculated for each group at 4 weeks and 8 weeks of intervention, 2 weeks and 8 weeks of post-intervention follow-up. Chi-square test is used to compare the differences in response rates between the groups, and logistic regression is used to calculate the relative risk (RR) and 95% confidence interval (95%CI).

(3) Serum indexes reflecting gastric digestibility

The change values of each index from baseline to 8 weeks of intervention (△1), and from 8 weeks of intervention to 2 weeks post the intervention (△2) are described respectively, and least-squares means and 95%CIs are calculated. Comparisons of △1 and △2 between groups are performed by independent t test.

Supplementary Reference

[1] Westermann, C., et al. A critical evaluation of *Bifidobacterial* adhesion to the host tissue. *Front Microbiol*, 2016,7:1220.

[2] Zhu, D., et al. Complete genome sequence of *Bifidobacterium animalis* subsp. *lactis* KLDS 2.0603, a probiotic strain with digestive tract resistance and adhesion to the intestinal epithelial cells. *J Biotechnol*, 2016,220:49-50.

[3] Sundararaman, A., et al. Genome of *Bifidobacterium longum* NCIM 5672 provides insights into its acid-tolerance mechanism and probiotic properties. *Arch Microbiol*, 2021,203(10):6109-6118.

[4] Wang, X., et al. Sodium oligomannate therapeutically remodels gut microbiota and suppresses gut bacterial amino acids-shaped neuroinflammation to inhibit Alzheimer's disease progression. *Cell Res*, 2019,29(10):787-803.

[5] Chen, S., et al. fastp: an ultra-fast all-in-one FASTQ preprocessor. *Bioinformatics*, 2018,34(17):i884-i890.

[6] Langmead, B., et al. Fast gapped-read alignment with Bowtie 2. *Nat Methods*, 2012,9(4):357-359.

[7] Beghini, F., et al. Integrating taxonomic, functional, and strain-level profiling of diverse microbial communities with bioBakery 3. *Elife*, 2021,10.

[8] Wang, X., et al. Aberrant gut microbiota alters host metabolome and impacts renal failure in humans and rodents. *Gut*, 2020,69(12):2131-2142.

[9] Tian, H., et al. Volatile organic compounds fingerprinting in faeces and urine of Alzheimer's disease model SAMP8 mice by headspace-gas chromatography-ion mobility spectrometry and headspace-solid phase microextraction-gas chromatography-mass spectrometry. *J Chromatogr A*, 2020,1614:460717.

[10] Zhao, L., et al. Gut bacteria selectively promoted by dietary fibers alleviate type 2 diabetes. *Science*, 2018,359(6380):1151-1156.

[11] Perry, R. J., et al. Acetate mediates a microbiome-brain-beta-cell axis to promote metabolic syndrome. *Nature*, 2016,534(7606):213-217.

[12] Ghoshal, U. C., et al. Development, translation and validation of enhanced Asian Rome III questionnaires for diagnosis of functional bowel diseases in major Asian languages: a Rome foundation-Asian neuro gastroenterology and motility association working team report. *J Neurogastroenterol Motil.* 2015,21:83-92.

[13] Tack, J., et al. Symptoms associated with hypersensitivity to gastric distention in functional dyspepsia. *Gastroenterol*. 2001,121:526-535.

[14] Tack, J., et al. Functional dyspepsia--symptoms, definitions and validity of the Rome III criteria. Nat. Rev. *Gastroenterol. Hepatol*. 2013,10:134-141.

[15] Ford, A. C., et al. Effect of dyspepsia on survival: a longitudinal 10-year follow-up study. Am. *J. Gastroenterol*. 2012,107:912-921.

[16] Lacy, B. E., et al. Functional dyspepsia: the economic impact to patients[J]. Aliment *Pharmacol Ther*, 2013,38:170-177.

[17] Talley, N. J., et al. Functional dyspepsia. *N. Engl. J. Med*. 2015,373:1853-1863.

[18] Camilleri, M., et al. Current management strategies and emerging treatments for functional dyspepsia. *Nat Rev Gastroenterol Hepatol*. 2013,10:187-194.

[19] Agah, S., et al. Systematic review with meta-analysis: Effects of probiotic supplementation on symptoms in functional dyspepsia. *J Funct Foods*, 2020,68:103902.

[20] Nakae, H., et al. Gastric microbiota in the functional dyspepsia patients treated with probiotic yogurt. *BMJ Open Gastroenterol*, 2016,3:e109.

[21] Sun, E., et al. Beverages containing *Lactobacillus paracasei* LC-37 improved functional dyspepsia through regulation of the intestinal microbiota and their metabolites. J Dairy Sci, 2021,104:6389-6398.

[22] Khoder, G., et al. Probiotics upregulate trefoil factors and downregulate pepsinogen in the mouse Stomach. Int J Mol Sci, 2019,20:3901.

[23] Lu, H., et al. Safety evaluation of *Bifidobacterium lactis* BL-99 and *Lacticaseibacillus paracasei* K56 and ET-22 *in vitro* and *in vivo*. Front. Microbiol. 2021,12:686541.

[24] Chen, M., et al. *Bifidobacterium lactis* BL-99 modulates intestinal inflammation and functions in zebrafish models. Plos One. 2022,17:e262942.

[25] Zhang, Q., et al. *In vitro* study of *Bifidobacterium lactis* BL-99 with fructooligosaccharide synbiotics effected on the intestinal microbiota. Front. Nutr. 2022,9:890316.

[26] Wauters, L., et al. Efficacy and safety of spore-forming probiotics in the treatment of functional dyspepsia: a pilot randomised, double-blind, placebo-controlled trial. Lancet Gastroenterol. Hepatol. 2021,6:784-792.
