# Supplementary material for: Efficacy of Bifidobacterium animalis subsp. lactis BL-99 in the treatment of functional dyspepsia: a randomized placebo-controlled clinical trial
Source: Nat Commun. 2024 Jan 3;15:227. doi: 10.1038/s41467-023-44292-x (PMC10764899; doi:10.1038/s41467-023-44292-x)
Supplement: Supplementary file 3 — Reporting Summary [file 41467_2023_44292_MOESM3_ESM.pdf]

nature portfolio

Corresponding author(s): Fazheng Ren

Last updated by author(s): Dec 4, 2023

Reporting Summary

Nature Portfolio wishes to improve the reproducibility of the work that we publish. This form provides structure for consistency and transparency in reporting. For further information on Nature Portfolio policies, see our Editorial Policies and the Editorial Policy Checklist.

Statistics

For all statistical analyses, confirm that the following items are present in the figure legend, table legend, main text, or Methods section.

n/a	Confirmed	
☐	☒	The exact sample size (*n*) for each experimental group/condition, given as a discrete number and unit of measurement
☐	☒	A statement on whether measurements were taken from distinct samples or whether the same sample was measured repeatedly
☐	☒	The statistical test(s) used AND whether they are one- or two-sided<br>*Only common tests should be described solely by name; describe more complex techniques in the Methods section.*
☒	☐	A description of all covariates tested
☒	☐	A description of any assumptions or corrections, such as tests of normality and adjustment for multiple comparisons
☐	☒	A full description of the statistical parameters including central tendency (e.g. means) or other basic estimates (e.g. regression coefficient) AND variation (e.g. standard deviation) or associated estimates of uncertainty (e.g. confidence intervals)
☐	☒	For null hypothesis testing, the test statistic (e.g. *F*, *t*, *r*) with confidence intervals, effect sizes, degrees of freedom and *P* value noted<br>*Give P values as exact values whenever suitable.*
☒	☐	For Bayesian analysis, information on the choice of priors and Markov chain Monte Carlo settings
☐	☒	For hierarchical and complex designs, identification of the appropriate level for tests and full reporting of outcomes
☐	☒	Estimates of effect sizes (e.g. Cohen's *d*, Pearson's *r*), indicating how they were calculated

Our web collection on statistics for biologists contains articles on many of the points above.

Software and code

Policy information about availability of computer code

Data collection	No software was used.
Data analysis	Statistical analyses were performed using SPSS Statistics version 24 (SPSS Institute, Chicago, IL, USA), capscale function and adonis function of the R vegan 2.6.4 package.

For manuscripts utilizing custom algorithms or software that are central to the research but not yet described in published literature, software must be made available to editors and reviewers. We strongly encourage code deposition in a community repository (e.g. GitHub). See the Nature Portfolio guidelines for submitting code & software for further information.

Data

Policy information about availability of data

All manuscripts must include a data availability statement. This statement should provide the following information, where applicable:

- Accession codes, unique identifiers, or web links for publicly available datasets
- A description of any restrictions on data availability
- For clinical datasets or third party data, please ensure that the statement adheres to our policy

The demographic characteristics, symptoms of functional dyspepsia, and serum markers data generated in this study are provided in the Supplementary Information/Source Data file. The Metagenomic sequencing data generated in this study have been deposited in the NCBI Sequence Read Archive (SRA) database

under the accession code PRJNA936638 [http://www.ncbi.nlm.nih.gov/bioproject/936638]. Mass spectral raw data generated in this study have been deposited in the MetaboLights database under the accession code MTBLS7169 [www.ebi.ac.uk/metabolights/MTBLS7169]. The clinical study protocol and statistical analysis plan file are provided in Supplementary Note 2 and Supplementary Note 3, respectively. The other data supporting the findings of this study are available within the paper and additional files. Source data are provided with this paper.

Research involving human participants, their data, or biological material

Policy information about studies with human participants or human data. See also policy information about sex, gender (identity/presentation), and sexual orientation and race, ethnicity and racism.

Reporting on sex and gender	We followed SAGER Guidelines.<br>The participants' gender in this study was determined based on self-report and that gender was considered for subgroup analysis in the study design.<br>Gender-based analyses have been reported in Methods, Results and Discussion sections.
Reporting on race, ethnicity, or other socially relevant groupings	NA
Population characteristics	Demographic and baseline characteristics of this study, including age, gender, body mass index (BMI), and symptom scores, are summarized in Table 1.<br>Between 26 December 2020 and 10 February 2021, 336 consecutive FD patients were screened and assessed for eligibility. A total of 123 individuals were excluded because they did not meet the inclusion criteria (n = 104), withdrew consent (n = 12), or had other reasons (n = 7). After enrolment, 13 patients were excluded due to withdrawal of consent (n = 8) or other reasons (n = 5), leaving a total of 200 patients who were then randomly assigned to four groups. Among these, 185 (92.5%) completed the entire clinical trial (45, 48, 47, and 45 in the placebo, positive_control, BL-99_low, and BL-99_high group, respectively; Fig. 1). Baseline characteristics are summarised in Table 1. The four treatment groups had similar baseline characteristics. The mean age of the participants was 51.43 years, the mean BMI was 25.24 kg/m2, and 74.5% were female.
Recruitment	Outpatients (18-60 years) with FD symptoms were recruited and screened between 26 December 2020 and 10 February 2021 at CCMU and CPLAGH.<br>This is a hospital-based study that recruited patients from outpatient clinics, the results may not be generalizable to the general FD population, such as those in the community. Besides, as only Chinese patients were recruited at the Beijing Chaoyang Hospital, Capital Medical University (CCMU; Beijing, China) and Chinese PLA General Hospital (CPLAGH; Beijing, China), the effectiveness of BL-99 in patients with FD from different countries, ethnicities, and clinical backgrounds was not evaluated.<br>All participants provided written informed consent before inclusion, and were compensated fairly in accordance with the requirements of the Institutional Review Board of CCMU, without any inducements.
Ethics oversight	This study was approved by the Institutional Review Board of Beijing Chaoyang Hospital, Capital Medical University (No.2020-ke-497) and was performed by the Declaration of Helsinki. The study was registered at Chictr.org.cn with a registration number of ChiCTR2000041430.

Note that full information on the approval of the study protocol must also be provided in the manuscript.

Field-specific reporting

Please select the one below that is the best fit for your research. If you are not sure, read the appropriate sections before making your selection.

[x] Life sciences [ ] Behavioural & social sciences [ ] Ecological, evolutionary & environmental sciences

For a reference copy of the document with all sections, see nature.com/documents/nr-reporting-summary-flat.pdf

Life sciences study design

All studies must disclose on these points even when the disclosure is negative.

Sample size	In a study of probiotics improving FD, the CRRs after 8 weeks of treatment of probiotic (5 × 109 CFU /day) and placebo were 48% and 20%, respectively, which were thus assumed for the low-dose probiotic group (1 × 1010 CFU/day, 48%) and the placebo group (20%) in our sample size calculation. In addition, we assumed a CRR of 50% for the positive drug group based on a study, and an intermediate value of 49% for the high-dose probiotic group, which was between the low-dose probiotic group and the positive drug group. Based on these assumptions, a sample size of 42 would be required per group (power of 80% and two-sided α = 0.05). Considering a 20% dropout rate, 50 subjects would be needed to be included in each group (200 for 4 groups).
Data exclusions	The main data set for efficacy analysis in this study was the ITT set, which included all participants who were randomized. In the ITT set, missing values for symptom scores were imputed based on the last observation carried forward method. Violations that significantly affect efficacy included (but were not limited to) the following: a. Received interference therapy after inclusion; b. Poor compliance (e.g., with follow-up visits less than 80% of the required number of visits); c. Follow-up beyond the window period44. We also analyzed symptom scores based on the PP set, which referred to participants who had completed the planned treatment and visits according to the protocol and had no obvious effect on the therapeutic effect.
Replication	This was a randomized clinical trial replicated in 50 independent participants per arm (200 total). The main data set for efficacy analysis in this

	study was the ITT set, which included all participants who were randomized. In the ITT set, missing values for symptom scores were imputed based on the last observation carried forward method.
Randomization	We used computer-generated random numbers to establish simple randomized grouping sequences. Eligible participants were identified by clinicians, and information was then transmitted via telephone, or email to a specialized statistician who had no further role in the trial to determine the treatment allocation based on the pre-established allocation sequence, which was concealed until all participants were allocated. Participants were randomly assigned (1: 1: 1: 1) to 4 groups, which included the placebo, positive_control (only PPI treatment), low-dose probiotic, and high-dose probiotic groups (only BL-99 treatment).
Blinding	Due to the difficulty of making probiotic formulations identical to PPI drugs, blinding was not possible in all four treatment groups. The positive-drug group was treated with PPI pills, and the other three groups received solid beverage powder with identical appearance, taste, and smell between groups. Therefore, the positive-drug group was open-label. For the other three groups, researchers and participants were blinded to treatment assignments until the study was completed.

Reporting for specific materials, systems and methods

We require information from authors about some types of materials, experimental systems and methods used in many studies. Here, indicate whether each material, system or method listed is relevant to your study. If you are not sure if a list item applies to your research, read the appropriate section before selecting a response.

Materials & experimental systems

n/a	Involved in the study
☒	☐ Antibodies
☒	☐ Eukaryotic cell lines
☒	☐ Palaeontology and archaeology
☐	☒ Animals and other organisms
☐	☒ Clinical data
☒	☐ Dual use research of concern
☒	☐ Plants

Methods

n/a	Involved in the study
☒	☐ ChIP-seq
☒	☐ Flow cytometry
☒	☐ MRI-based neuroimaging

Animals and other research organisms

Policy information about studies involving animals; ARRIVE guidelines recommended for reporting animal research, and Sex and Gender in Research

Laboratory animals	Thirty-six normal male Sprague-Dawley rats (8-9 weeks of age, 350-390 g) were ordered from Charles River Laboratories (CRL, Beijing, China).
Wild animals	This study did not include wild animals.
Reporting on sex	Male rats were used for the experiments. Males were chosen because the larger vessel size of male rats makes the experimental model more reliable. We do not expect the findings will be sex-specific.
Field-collected samples	No field-collected samples were used.
Ethics oversight	All the experimental procedures were approved by the Ethics Committee of Beijing Laboratory Animal Research Center (approval No. BLARC-LAWER-202306006), and were also in accordance with the Guidelines for the Care and Use of Laboratory Animals published by the US National Institutes of Health.

Note that full information on the approval of the study protocol must also be provided in the manuscript.

Clinical data

Policy information about clinical studies

All manuscripts should comply with the ICMJEguidelines for publication of clinical research and a completedCONSORT checklist must be included with all submissions.

Clinical trial registration	Chinese Clinical Trial Registry, ChiCTR2000041430
Study protocol	The study procedures are shown in Supplementary Fig. 7.<br>All included participants first underwent a 2-week run-in period. During the run-in period, participants were not allowed to take foods containing probiotics (such as probiotic powder, probiotic yogurt, etc.). Then participants were treated with PPI, BL-99, or placebo for 8 weeks, followed by an 8-week post-treatment follow-up. During the treatment, participants were instructed to maintain their habitual lifestyle habits such as diet and physical activity and were not allowed to take antibiotics.
Data collection	A total of 4 visits [at baseline (V1), 4-week treatment (V2), 8-week treatment (V3), and 2-week follow-up (V4)] and 1 survey [questionnaire surveys 8 weeks after the treatment (V5)] were conducted throughout the study period. At each visit and the final survey, participants were surveyed using a uniform FD symptom assessment questionnaire (see the 'Symptom assessment' section

	for details). Blood and fecal samples were collected at V1, V3, and V4. Even 8 weeks post the treatment, we were fortunate to receive the questionnaire responses from all participants who completed the treatment.
Outcomes	The primary outcome was the clinical response rate (CRR) of the FD score at week 8 of treatment. Clinical response was defined as a score (i.e., FD score, PDS score, and EPS score) decrease > 0.5. CRR was then calculated as the proportion of clinical responders13. The secondary endpoints were CRR of FD score at week 4 of treatment, week 2 and 8 of follow-up; CRR of PDS score and EPS score at every visit or survey after initiation of treatment; changes in serum indicators (PG Ⅰ, PG Ⅱ, PGR, and G17), fecal microbiota, fecal metabolites, and changes of SCFA in feces and serum from baseline to 8-week treatment and 2-week follow-up periods.<br>Subjects were asked to report any adverse effects during the treatment and follow-up periods, such as bloating, nausea, diarrhea, itchy skin, etc. Safety was assessed by classifying adverse events using the Common Terminology Criteria for Adverse Events (CTCAE) version 5.0 at each study period or in the case of early termination.

Plants

Seed stocks	NA
Novel plant genotypes	NA
Authentication	NA
